# Complete Coding Sequence of a Chikungunya Virus Strain Imported into Slovenia from Thailand in Late 2018

**DOI:** 10.1128/MRA.00581-19

**Published:** 2019-09-12

**Authors:** Samo Zakotnik, Misa Korva, Nataša Knap, Barbara Robnik, Nina Gorišek Miksić, Tatjana Avšič Županc

**Affiliations:** a Institute of Microbiology and Immunology, Faculty of Medicine, University of Ljubljana, Ljubljana, Slovenia; b Department of Infectious Diseases, University Medical Centre Maribor, Maribor, Slovenia; KU Leuven

## Abstract

A case of chikungunya virus infection was imported from Thailand into Slovenia in late 2018. The infection was diagnosed using real-time reverse transcription-PCR, the virus was isolated in cell culture, and the whole genome was sequenced. Phylogenetic analysis of the nearly complete viral genome indicated that the virus belongs to the Indian Ocean lineage but does not possess the A226V mutation in the envelope protein E1.

## ANNOUNCEMENT

Chikungunya virus (CHIKV) belongs to the Alphavirus genus of the *Togaviridae* family, and, similar to other arthritogenic alphaviruses, the infection is characterized by acute fever that progresses to severe and persistent arthralgia. The disease is mostly self-limiting, but in some patients, debilitating joint pain can persist for years (1). Chikungunya is a vector-borne virus that can be transmitted by the Aedes aegypti or Aedes albopictus vector (2).

The genome of CHIKV is a single-stranded positive-sense RNA and is approximately 12 kb long. It encodes 4 nonstructural proteins (nsP1 to -4) and 5 structural proteins (C, E3, E2, 6K, and E1). It was first isolated in 1952 in Tanzania (2). The most recent outbreak of CHIKV started in Thailand in October 2018. By the end of the year, the Thai Ministry of Public Health reported more than 2,000 cases of chikungunya infection. The outbreak is still ongoing, with more than 1,500 cases reported in January and February 2019 (3). Until now, at least 9 chikungunya cases were imported to Europe, specifically to Sweden, Switzerland, the United Kingdom, Romania, Italy, and France (3).

In November 2018, a 38-year-old male patient returned from a 21-day trip through Thailand suffering from an acute febrile illness, manifesting with fever and chills, severe arthralgia, headache, occasional dry cough, and gastrointestinal symptoms. He was ill for 1 day. Inflammatory markers were consistent with a viral disease with a C-reactive protein level of 68 mg/liter, a normal leukocyte count, and mildly elevated liver transaminase enzyme levels. He reported a mosquito bite with a local reaction, followed by a macular rash over his trunk 4 to 5 days prior to the acute onset of fever. Real-time reverse transcription-PCR demonstrated the presence of CHIKV RNA (4) in a blood sample obtained at hospitalization (i.e., second day of fever), and the diagnosis was confirmed with virus isolation on the Vero E6 cell line and genome sequencing.

For sequencing, supernatant was collected from the Vero E6 cell culture 3 days after inoculation. Prior to RNA isolation, the supernatant was centrifuged (4°C, 10 min, 12,000 × *g*) and filtered through a 0.2-μm filter. RNA was extracted with TRIzol and the Direct-zol RNA miniprep kit (Zymo Research), and DNA was removed with DNase I, following the manufacturer’s instructions. cDNA was synthesized with the Maxima H minus double-stranded cDNA synthesis kit (Thermo Fisher Scientific) using random primer A (5′-GTTTCCCAGTCACGATCNNNNNNNNN-3′) instead of random hexamers and further amplified with PCR amplification using primer B (5′-GTTTCCCAGTCACGATC-3′), according to the SISPA protocol (5). A next-generation sequencing (NGS) library was constructed using the Nextera XT kit, and samples were labeled with the Nextera XT index kit, according to the manufacturer’s instructions (Illumina). Libraries were sequenced using the MiSeq reagent kit V3 and the MiSeq system. Sequencing generated more than 3 × 105 reads (paired at 2 × 301 nucleotides [nt]). Data were filtered, adapter sequences were removed with Trimmomatic (default settings, Illuminaclip was performed with in-built adapter sequences for Nextera paired-end libraries) (6), and the genome was *de novo* assembled using the Unicycler (default settings) (7) on the Galaxy server (8). The average coverage of the final contig is 5,635×, and the GC content is 50.04%. The contig origin was determined by a BLAST search. In comparison to the most similar sequence of CHIKV (GenBank accession number MH400249), the newly assembled genome is 666 nt shorter on the 5′ end and 56 nt shorter on the 3′ end.

The assembled genome is 11,063 nt long. Open reading frames (ORFs) were found using the NCBI ORFfinder (minimal ORF length set to 600 nt) (9) and manually annotated according to already-annotated sequences of CHIKV found in the UniProt database. The first ORF encodes a 2,247-amino-acid (aa)-long nonstructural polyprotein that is processed in a cell to proteins ns1, ns2, ns3, and ns4. The genome is missing an opal readthrough stop codon between Leu-1629 and Leu-1631 (between ns3 and ns4) and has instead arginine as the 1,630th amino acid of the nonstructural polyprotein. The opal readthrough stop codon between ns3 and ns4 regulates the transcription of ns4. CHIKV circulates in two variants, one with an opal termination codon and the second with an opal codon readthrough. Reports show that the opal codon is important for viral maintenance in vertebrate and invertebrate hosts and that a selective advantage is conferred in Vero cells for the sense (arg) codon (10).

The second ORF is a 1,248-aa-long structural polyprotein and codes for proteins C, E3, E2, 6K, and E1. The ORF does not contain a mutation at the position 226 on the E1 glycoprotein (E1-A226V). This mutation was associated with enhanced transmission by *A. albopictus* mosquitos in regions where the major mosquito vector, A. aegypti, is absent (11). Such mutation and consequent vector switching could be the cause of the recent spike in CHIKV infections in southern Thailand, but since it is absent from the genome, the underlying cause of the CHIKV spread remains unknown.

**FIG 1:**
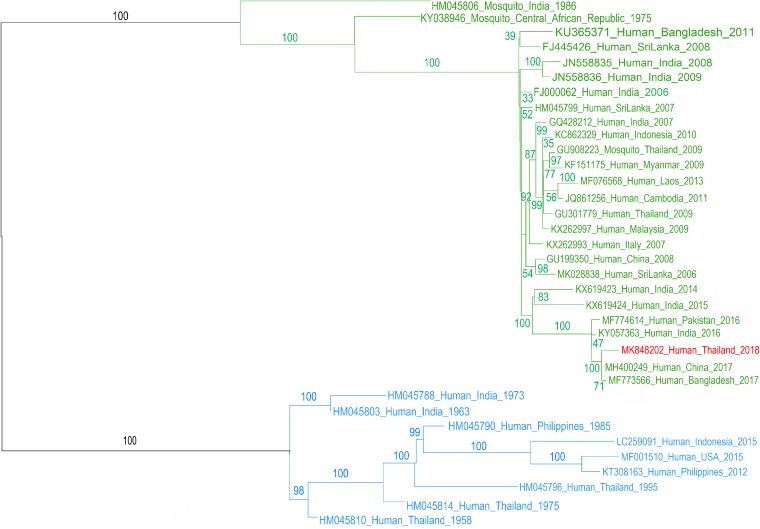
The phylogenetic tree of 35 sequences of CHIKV chosen from the NCBI. The sequences belong to two lineages, the Indian Ocean lineage (green) and the Asian and Caribbean lineage (blue). The genome sequence of CHIKV imported to Slovenia (GenBank accession number MK848202, red) is part of the Indian Ocean lineage and most closely related to the sequence from China (GenBank accession number MH400249). Labels on the branches show bootstrap values.

The phylogenetic analysis (Fig. 1) indicates that the virus belongs to the Indian Ocean lineage and that it associates with a recent isolate from China (GenBank accession number MH400249) with 99.8% nucleotide identity, based on a best blastn hit (Web BLAST). The alignment of selected genomic sequences was made using the MUSCLE alignment algorithm (12) in the UNIPRO Ugene (version 1.32, default settings) (13), and the phylogenetic analysis was made with IQ-Tree (version 1.6.10 using 100 bootstrap replicates) (14, 15). The phylogenetic tree was drawn with FigTree (version 1.4.4).

### Data availability.

The genome sequence was deposited in GenBank under accession number MK848202. The raw reads were deposited into the SRA under accession number SRR9077329.
